# Social exclusion and suicide intention in Chinese college students: a moderated mediation model

**DOI:** 10.3389/fpsyg.2024.1354820

**Published:** 2024-02-02

**Authors:** Juncheng Zhu, Pei Xie, Xingyu Zhang

**Affiliations:** Hunan Key Laboratory of Children’s Psychological Development and Brain Cognitive Science, Department of Education, Hunan Frist Normal University, Hunan, Changsha, China

**Keywords:** social exclusion, meaning in life, depression, suicidal ideation, college

## Abstract

Given the growing incidence rates of suicide among college students and the potential lifelong consequences of suicide, it is imperative to better understand the factors that reduce the rates at which college students in a clinical sample engage in suicide. This study examines the relationship between social exclusion and suicide intention, the mediating effect of depression, and the moderating effect of meaning in life. Two hundred and ninety-nine Chinese college students, aged from 18 to 22 years (56.86% female, *M* age = 20.14, SD = 1.27) completed questionnaires assessing their social exclusion, suicide intention, depression, and meaning in life. The result revealed that social exclusion was positively associated with suicide intention, and depression mediated this relationship. In addition, this mediating effect of depression was moderated by meaning in life. That is, the mediation effect was stronger for students with a higher level of meaning in life. These findings provide educational suggestions for preventing and intervening in suicide intention among college students.

## 1 Introduction

Suicide is a serious public safety issue that has long been a focus of researchers. According to a survey by the World Health Organization, suicide is the fourth leading cause of death among individuals aged 15–29 globally (World Health Organization, 2021), and this age group, particularly university students, requires special attention. Research has shown that suicide accounts for the largest proportion of non-natural deaths reason (including causes such as illness, accidents, homicides, suicides, and unknown reasons) among Chinese university students (Yang and Li, 2015). The most significant precursor to suicidal behavior is suicidal ideation (Li et al., 2014). In the age group of university students, approximately 3.5–7% of individuals in China have experienced suicidal thoughts or tendencies (Li et al., 2012). Suicidal ideation refers to the cognitive or thought process of considering suicide or self-harm (Murrough et al., 2015), including but not limited to specific death plans and explicit suicidal intentions (Jobes and Joiner, 2019). Therefore, studying the influencing factors and mechanisms of suicidal ideation among university students is of great practical significance.

Many previous studies have explored the causes of suicide from aspects such as personality traits, depressive emotions, and mental health (Gvion and Levi-Belz, 2018), while relatively few have focused on interpersonal relationships and chronic social trauma. Social exclusion often occurs in interpersonal interactions and belongs to a form of chronic social trauma. Prolonged experiences of social exclusion can lead to varying degrees of thwarted belongingness and perceived burdensomeness (Fu, 2021). The interpersonal theory of suicide proposes that one of the underlying causes of suicidal ideation may be poor interpersonal states (Van Orden et al., 2010; Christensen et al., 2014). For example, when individuals experience long-term external rejection or a lack of belongingness, they are more likely to develop depressive emotions and a sense of meaninglessness, leading to suicidal ideation and even suicidal behavior (Joiner, 2007). Previous studies have examined groups such as bereaved parents and left-behind children, and the results have shown that the more social exclusion they experience, the higher their suicidal ideation (Wang et al., 2022). This indicates that social exclusion can influence suicidal ideation, but it is still unclear which factors affect the relationship between the two. Therefore, this study aims to investigate the relationship between social exclusion and suicidal ideation among university students, as well as potential mediating or moderating variables. This research will provide important theoretical foundations for the prevention and intervention of suicidal ideation among university students to some extent.

### 1.1 Social exclusion and suicidal ideation

Social exclusion refers to the rejection or exclusion individuals experience from groups or individuals during interpersonal interactions, leading to a lack of fulfillment of their sense of belongingness (MacDonald and Leary, 2005). Social exclusion can manifest in various forms, such as isolation, neglect, and rejection, which affect the formation of stable relationships with others. However, being accepted by certain social groups and forming positive, stable, and enduring social relationships with others is one of the most fundamental and universal needs for humans. Many aspects of an individual’s daily activities and well-being depend on their sense of belongingness to a group and others (Du and Xia, 2008). According to Maslow’s hierarchy of needs theory, a sense of belongingness is a basic human need (Beaumeister and Leary, 1995; Holt-Lunstad et al., 2015), and the lack of it may lead to a range of health risks (Cacioppo and Cacioppo, 2014), including suicidal ideation and even suicidal behavior (Turecki and Brent, 2016). At the same time, researchers believe that individuals who are excluded from a group due to various reasons may experience a lack of interpersonal relationships, which can have negative impacts on them (Zhou, 2004). Particularly, individuals who are sensitive to interpersonal relationships perceive higher levels of exclusion, which can strengthen their suicidal ideation (Albanese et al., 2021). The interpersonal theory of suicide suggests that when individuals experience thwarted belongingness and perceived burdensomeness, they develop suicidal ideation. Both thwarted belongingness and social exclusion impact an individual’s sense of belongingness and are important factors contributing to suicidal ideation (Joiner, 2007; Van Orden et al., 2010). The aforementioned findings indicate that social exclusion may be an important predictive factor for suicidal ideation.

### 1.2 Mediating role of depression

Depression is one of the most common symptoms of mental health problems. According to the World Health Organization’s 2023 report on “Depressive Disorders (Depression),” approximately 4% of the global population suffers from varying degrees of clinical depression, making it a prevalent mental illness worldwide (World Health Organization, 2023). In individuals with mild depression, it is a transient emotional experience that can affect their interpersonal interactions, sleep patterns, and other aspects of their lives. However, for individuals with more severe depression, it can significantly impact their self-perception and cognitive abilities. According to the escape theory of suicide (Baumeister, 1990), suicide can be seen as an impulsive decision made by individuals facing overwhelming emotional distress, seeking to alleviate their current psychological pain by ending their lives (Klonsky et al., 2016). If the level of depression continues to escalate, self-harm or suicidal behavior may occur (Conejero et al., 2018; Shumye et al., 2019). Additionally, social exclusion is closely associated with the occurrence of depression (Niu et al., 2022). Social exclusion can trigger psychological and physiological pain, leading to anxiety and depressive symptoms (Bungert et al., 2015). Numerous studies have also found that individuals who experience social exclusion exhibit more depressive symptoms (Baumeister, 1990; Niu et al., 2016; Li et al., 2018). Slavich et al. (2010) proposed a psychobiological model suggesting that stressors related to exclusion can trigger a range of unique and integrated cognitive, emotional, and biological changes that may lead to depression. In this model, social exclusion events activate brain regions associated with processing negative emotions and distress related to exclusion (such as the anterior insula and dorsal anterior cingulate cortex), which can also trigger negative self-referential cognitions and associated negative self-awareness emotions, potentially leading to suicidal ideation or behavior. Based on this theoretical and empirical evidence, it is hypothesized that depression could mediate the relationship between social exclusion and suicidal ideation.

### 1.3 Moderating role of meaning in life

**Figure 1:**
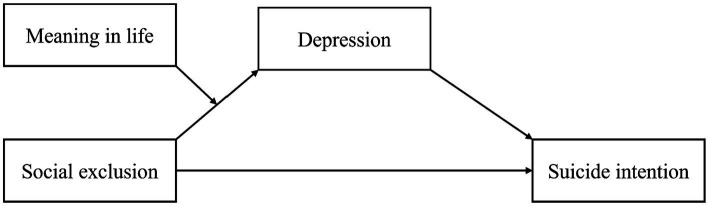
The hypothesized model.

Meaning in life refers to an individual’s perception and understanding of the meaning of their life, the search for life goals and missions, and the subjective sense of the importance and purpose of one’s existence (Steger et al., 2006). This capacity for perception and awareness involves an individual’s understanding and identification of the purpose and value of their existence, and it is the subjective experience of the importance and purpose of one’s existence. Therefore, a decrease in meaning in life may lead to a range of psychological health problems, including depression, anxiety, hopelessness, and suicidal crisis (Glaw et al., 2017; Arslan and Yıldırım, 2021). Furthermore, while meaning in life is not explicitly or inherently dependent on social relationships, individuals are likely to find meaning in their social relationships in practice (Stillman et al., 2009). This indirectly suggests that individuals need validation from others, and when individuals experience problems in their interpersonal relationships due to social exclusion, their perception of meaning in life may play a significant role. Research has shown that an individual’s perception of meaning in life is a powerful psychological resilience factor (Gallagher and Miller, 2018). Meaning in life has been demonstrated to be a protective factor against depression, despair, and suicidal ideation (Sun et al., 2022). This is because meaning in life can buffer and mitigate the negative impact of stressful events, thereby avoiding impulsive behaviors and effectively reducing suicide risk (Kleiman et al., 2013; Costanza et al., 2019). It can be inferred that when individuals recognize their life value and meaning, they may rationally face the negative impact of events such as social exclusion without experiencing depressive emotions or even suicidal ideation or behavior. Therefore, this study suggests that meaning in life may moderate the first half of the “social exclusion → depression → suicide ideation” mediating pathway (Figure 1).

### 1.4 Participants

The participants in this study were undergraduate students. A total of 318 questionnaires were collected, with 19 regular response patterns excluded. All participants were recruited randomly (through convenience sampling). The final valid sample consisted of 299 participants, resulting in a questionnaire validity rate of 94.03%. Among the participants, there were 170 females (56.86%) and 129 males (43.14%). The average age of the participants was 20.14 years (*SD* = 1.27), with ages ranging from 18–22 years.

## 2 Methods

### 2.1 Social exclusion scale

The social exclusion scale (OES-A) was used, which was developed by Gilman et al. (2013) and translated into Chinese by Zhang et al. (2018). The Cronbach coefficients of the Chinese version of the scale was 0.82 (Zhang et al., 2018). The scale consists of 11 items, such as “Others often come to my house to play with me.” It assesses two dimensions: neglect and rejection. A five-point scoring system was used, with 1 indicating “never” and 5 indicating “always.” Higher scores indicate a higher degree of social exclusion. The Social Exclusion Scale has demonstrated good reliability and validity in previous studies with adolescents (Zhang et al., 2018). In this study, Cronbach’s ⍺ coefficients for neglect and rejection were 0.900 and 0.905, respectively. The reliability of the social exclusion scale was 0.945.

### 2.2 Meaning in life questionnaire

The meaning in life questionnaire (MLQ), developed by Steger et al. (2006) and revised by Wang (2013), was used. The Cronbach coefficients of the Chinese version of the scale was 0.83 (Wang, 2013). The questionnaire includes two subscales: Presence of Meaning (living a meaning) and Search for Meaning (perceived meaning). It consists of 10 items, with 5 items for each subscale. A seven-point rating scale was used, with 1 indicating “completely not true” and 7 indicating “completely true.” The original authors of the MLQ (Steger et al., 2006) validated its reliability and validity in a sample of college students. In this study, Cronbach’s ⍺ coefficients for presence of meaning and search for meaning were 0.928 and 0.924, respectively. The reliability of the meaning in life questionnaire was 0.962.

### 2.3 The patient health questionnaire-9

The patient health questionnaire-9 (PHQ-9) was used to assess depression severity, which is based on the DSM-IV criteria for depression (Kroenke et al., 2001). The instructions state, “Over the past 2 weeks, how often have you been bothered by the following problems? Please select the answer that best describes how you have been feeling in the past 2 weeks.” Items include statements such as “Feeling down, depressed, or hopeless.” The questionnaire consists of 9 items, rated on a 4-point scale (0–3), with 0 indicating “not at all” and 3 indicating “nearly every day.” the Cronbach coefficients of the Chinese version of the scale was 0.892 (Sun et al., 2020). The PHQ-9 has been widely used in Chinese adolescent populations and has demonstrated good reliability and validity (Wang et al., 2014). In this study, the reliability of the PHQ-9 Scale was 0.982.

### 2.4 Suicide ideation scale

The suicide ideation scale (PANSI), developed by Osman et al. (2002) and revised by Wang et al. (2011), was used. The Cronbach coefficients of the Chinese version of the scale was 0.92 (Wang et al., 2011). This scale consists of 14 items, divided into two dimensions: (1) Positive Suicide Ideation, which includes 6 items, such as “Feeling that most situations in life are under my control”; (2) Negative Suicide Ideation, which includes 8 items, such as “Feeling hopeless about the future and having thoughts of ending my life.” Each item is rated on a 1 to 5 scale, with 1 indicating “never” and 5 indicating “always.” The scores of the 6 items in the Positive Suicide Ideation dimension are reverse-scored and then summed with the scores of the 8 items in the Negative Suicide Ideation dimension to obtain a total score. Higher scores indicate a higher level of suicide ideation. The Suicide Ideation Scale has demonstrated good reliability and validity in practical applications with adolescent populations (Osman et al., 2002). In this study, Cronbach’s ⍺ coefficients for positive and negative suicide ideation were 0.895 and 0.960, respectively. The reliability of the Suicide Ideation Scale was 0.963.

## 3 Results

### 3.1 Test of common method bias

A Harman’s single-factor test was conducted to examine the potential common method bias by performing an exploratory factor analysis (unrotated) on all the items (Zhou and Long, 2004). The results revealed that there were four factors with eigenvalues >1. However, the first common factor accounted for only 18.87% of the cumulative variance, which is below the critical threshold of 40%. This indicates that the study is not significantly affected by common method bias.

### 3.2 Descriptive statistics and correlation analysis

The descriptive statistics and correlation analysis of the variables are presented in Table 1. The correlation analysis results indicate significant correlations between age, sex, social exclusion, meaning in life, depression, and suicide ideation.

**Table 1:** Descriptive statistics and correlation matrix for the study variable (*N* = 299).

Variable	*M*	*SD*	1	2	3	4	5	6
1. Social exclusion	2.39	1.23	1					
2. Depression	2.26	1.74	0.36^**^	1				
3. Suicide intention	2.16	1.17	0.48^***^	0.64^**^	1			
4. Meaning in life	4.67	1.26	−0.42^***^	−0.35^***^	−0.49^***^	1		
5. Age	20.14	1.27	−0.01	−0.03	0.02	−0.02	1	
6. Sex	-	-	−0.10	−0.16^**^	−0.06	−0.04	0.04	1

^**^p < 0.01, ^***^p < 0.001, gender was dummy coded such that 1 = male, 2 = female.

### 3.3 Relationship between social exclusion and suicide ideation: moderated mediation analysis

All data were analyzed using SPSS 19.0 and the PROCESS macro developed by Hayes (2013). The simple mediation model and moderated mediation model were tested using the built-in Model 4 and Model 7 in PROCESS (Hayes, 2013; Wen and Ye, 2014). The results are presented in Table 2.

**Table 2:** Results of moderated mediation model.

Predictors	Depression (first step)			Suicide intention (first step)			Depression (second step)		
	*β*	*SE*	*t*	*β*	*SE*	*t*	*β*	*SE*	*t*
Social exclusion	0.13	0.02	6.55^***^	0.30	0.05	6.48^***^	0.12	0.02	5.31^***^
Depression				1.48	0.12	11.95^***^			
Meaning in life							−0.07	0.01	−4.70^***^
Social exclusion × Meaning in life							0.05	0.01	3.72^***^
*R^2^*		0.35			0.69			0.46	
*F*		42.93^***^			137.34^***^			26.47^***^	

In the first step, the simple mediation model was examined. Regression analysis revealed that social exclusion significantly predicted suicide ideation positively (*β* = 0.48, *p* < 0.001). After including depression in the regression equation, social exclusion still had a significant positive effect on suicide ideation (*β* = 0.30, *p* < 0.001). Social exclusion positively predicted depression (*β* = 0.13, *p* < 0.001), and depression positively predicted suicide ideation (*β* = 1.48, *p* < 0.001). The indirect effect (ab) was 0.20, *Boot SE* = 0.04, and the 95% confidence interval was [0.13, 0.27]. This indicates that depression significantly mediated the relationship between social exclusion and suicide ideation, accounting for 39.60% of the total effect.

In the second step, the moderated mediation model was examined. The results showed that social exclusion positively predicted depression (*β* = 0.12, *p* < 0.001), while meaning in life negatively predicted depression (*β* = −0.07, *p* < 0.001). Additionally, the interaction between social exclusion and meaning in life significantly predicted depression (*β* = 0.05, *p* < 0.001), with a 95% confidence interval of [0.02, 0.07]. This indicates a significant moderating effect of meaning in life. These results suggest that meaning in life plays a moderating role in the first half of the mediation path “social exclusion → depression → suicide ideation.”

**Figure 2:**
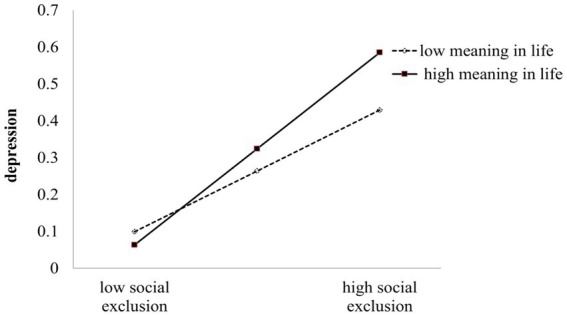
Map of moderating effects of meaning in life.

To understand how meaning in life moderates the relationship between social exclusion and depression, we conducted further simple effects analysis. Meaning in life was grouped into high and low levels based on one standard deviation above and below the mean. The results revealed that when individuals had a higher sense of meaning in life (+1 SD), social exclusion had a significant positive predictive effect on depression (*b*_simple_ = 0.28, *p* < 0.01), with a 95% confidence interval of [0.19, 0.38]. Conversely, when individuals had a lower sense of meaning in life (−1 SD), social exclusion still had a significant positive predictive effect on depression (*b*_simple_ = 0.07, *p* < 0.05), with a 95% confidence interval of [0.003, 0.14] (Figure 2).

## 4 Discussion

This study reveals the relationship between social exclusion and suicidal ideation among college students, as well as the underlying mechanisms involved. On one hand, it elucidates how social exclusion operates by influencing suicidal ideation through the mediating role of depression. On the other hand, it examines when the effect is stronger, highlighting the moderating role of meaning in life, which manifests as a stronger mediating effect of depression in the high meaning in life.

### 4.1 The relationship between social exclusion and suicidal ideation among college students

Social exclusion has a significant positive predictive effect on suicidal ideation, indicating that the higher the degree of social exclusion an individual experiences, the stronger their suicidal ideation. This finding is consistent with numerous previous studies (Gao et al., 2010; Xue et al., 2022). It further corroborates the interpersonal theory of suicide, which suggests that when individuals experience rejection or isolation from a group, their need for belongingness goes unfulfilled, making them more susceptible to suicidal thoughts (MacDonald and Leary, 2005). Wang et al. (2023) also found that individuals who experience prolonged social exclusion not only feel lonely but also perceive themselves as unwanted and worthless, which contributes to increased suicidal ideation. Therefore, social exclusion serves as an important risk indicator for suicidal ideation and even suicidal behavior among college students. This highlights the significance of addressing social exclusion and enhancing students’ sense of belongingness in promoting their mental well-being and reducing suicidal ideation.

### 4.2 The mediating role of depression in the relationship between social exclusion and suicidal ideation among college students

This study found that social exclusion influences suicidal ideation through the pathway of depression. Specifically, social exclusion makes individuals more susceptible to experiencing depressive emotions, which in turn leads to suicidal ideation. Our findings support the psychobiological model (Slavich et al., 2010), which suggests that socially rejecting life events trigger a cascade of reactions that result in individuals developing negative self-awareness emotions (such as depression) or behavioral issues (Wang et al., 2019). Social exclusion, being a relatively common life event, elicits a range of negative emotions in individuals, including feelings of loneliness, depression, anxiety, and helplessness (Heffer and Willoughby, 2018). College students, as independent individuals, are at an age where they particularly need a sense of belongingness. When faced with social exclusion from peers or groups, they are more prone to experiencing negative emotions and getting trapped in a cycle of negativity. According to the escape theory of suicide, prolonged negative emotions can lead to immense psychological distress, making individuals more susceptible to suicidal thoughts (Baumeister, 1990). Thus, the psychological distress caused by depression serves as a bridge between social exclusion and suicidal ideation among college students.

### 4.3 The moderating role of meaning in life in the mediating effect of depression

This study revealed, for the first time, that meaning in life plays a moderating role in the first half of the mediation effect of “social exclusion → depression → suicidal ideation.” Specifically, compared to individuals with low meaning in life, social exclusion has a stronger predictive effect on depression for individuals with high meaning in life. This finding is consistent with previous research (Stillman et al., 2009), which found a close relationship between high levels of search for meaning (one dimension of meaning in life) and low levels of negative functioning (e.g., suicidal ideation, depression, anxiety) (Mascaro and Rosen, 2006). It suggests that individuals with higher levels of meaning in life are more likely to experience more depression after experiencing social exclusion, which may lead to suicidal ideation or even suicidal behavior. Additionally, this study found an interesting result that, compared to individuals with high meaning in life, the predictive effect of social exclusion on depression is weaker for individuals with low meaning in life. This result aligns with the “the more lice, the less it itches” model proposed by Li (2012). This model suggests that when individuals experience negative emotions influenced by external factors, the presence of additional negative factors may lead to a feeling of “the more lice, the less it itches,” thereby reducing the impact between the first two factors. In this study, low meaning in life does not enhance the effect of social exclusion on depression; instead, it may “weaken” the effect of social exclusion on depression. However, this moderation pattern does not imply that meaning in life is a protective factor for depression, as individuals with low meaning in life generally have higher levels of depression compared to those with high meaning in life (Bonebright et al., 2000). The reason for this moderation pattern may be that individuals with low meaning in life have a higher susceptibility to depression, and their depression levels are already close to saturation, making it difficult for the predictive effect of social exclusion on depression to manifest.

### 4.4 Implications

On a theoretical level, this study demonstrates the direct relationship between social exclusion and suicidal ideation, providing an application and validation of the psychobiological model in a sample of Chinese college students. Furthermore, the study reveals the mediating and moderating effects of meaning in life and depression in the relationship between social exclusion and suicidal ideation, achieving integration across multiple models. On a practical level, the findings of this study contribute to considering strategies for preventing and addressing social exclusion to prevent suicidal ideation or suicidal behavior among college students. Specifically, social exclusion serves as an important risk indicator for suicidal ideation and even suicidal behavior among college students. This highlights the significance of addressing social exclusion and enhancing students’ sense of belongingness in promoting their mental well-being and reducing suicidal ideation. Future research can focus on disseminating knowledge about the harms of social exclusion, enhancing a sense of belongingness, and promoting positive lifestyles and coping strategies through mental health programs in universities to improve the mental well-being of college students.

### 4.5 Limitations and future directions

Undoubtedly, the current study has some limitations. First, this study could not establish the causal relationship between social exclusion and suicide intention using a cross-sectional study, so, future research can adopt longitudinal studies to cover the causal relationships (Maxwell et al., 2011). Second, the sampling of this study was limited and it did not examine the differences in the relationship between variables in grades and regions. In subsequent studies, we will expand the sampling scope to enhance the generalizability of the research conclusions. Third, this study only examines the relationship between social exclusion and suicide intention, the mediating effect of depression, and the moderating effect of meaning in life. Future research may consider other variables (e.g., precipitating entrapment, Giotakos, 2022; thwarted belongingness, Albanese et al., 2021) to further explore the relationship between social exclusion and suicide intention.

## Data availability statement

The datasets presented in this study can be found in online repositories. The names of the repository/repositories and accession number(s) can be found at: https://www.jianguoyun.com/p/DdTruVIQ3Z2dDBiKjqwFIAA.

## Ethics statement

The studies involving humans were approved by Ethics Committee of Hunan First Normal University. The studies were conducted in accordance with the local legislation and institutional requirements. The participants provided their written informed consent to participate in this study. Written informed consent was obtained from the individual(s) for the publication of any potentially identifiable images or data included in this article.

## Author contributions

JZ: Conceptualization, Data curation, Funding acquisition, Writing – original draft, Writing – review & editing. PX: Data curation, Formal analysis, Methodology, Validation, Writing – review & editing. XZ: Data curation, Formal analysis, Methodology, Resources, Validation, Writing – review & editing.
